# Correction: Alhajlah et al. Overexpression of Reticulon 3 Enhances CNS Axon Regeneration and Functional Recovery After Traumatic Injury. *Cells* 2021, *10*, 2015

**DOI:** 10.3390/cells15060556

**Published:** 2026-03-20

**Authors:** Sharif Alhajlah, Adam M Thompson, Zubair Ahmed

**Affiliations:** 1 Neuroscience and Ophthalmology, Institute of Inflammation and Ageing, University of Birmingham, Birmingham B15 2TT, UK; alhjlah@su.edu.sa (S.A.); a.thompson@exeter.ac.uk (A.M.T.); 2 Applied Medical Science College, Shaqra University, P.O. Box 1678, Ad-Dawadmi 11911, Saudi Arabia; 3 Centre for Trauma Sciences Research, University of Birmingham, Edgbaston, Birmingham B15 2TT, UK

In the original manuscript [1], there was a mistake in Figure 8 as published. In the original Figure 8G, the ONC+shEGFP and ONC+RTN3+shProtrudin panels were similar to the PBS and PEDF panels of another published article [2]. When compiling the figure, the wrong raw image was copied over from the storage device and used in the figure. The corrected Figure 8 appears below.

Additionally, the Conflicts of Interest statement was updated for full transparency. The corrected statement is below.

## Figures and Tables

**Figure 8:**
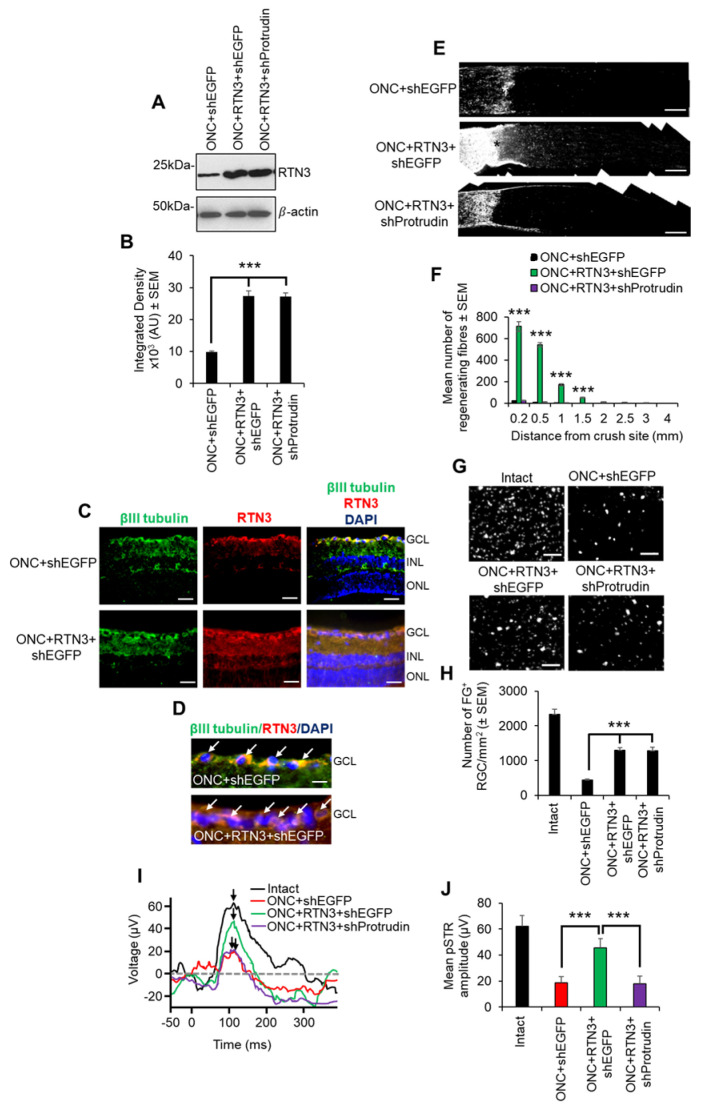
RTN3 overexpression promotes RGC axon regeneration and functional recovery after ONC, which is also protrudin-dependent. (**A**) Western blot and (**B**) quantification to show that RTN3 plasmids significantly upregulate RTN3 protein in the retina after intravitreal injection in vivo. (**C**) Immunohistochemistry to localise RTN3 protein to the RGCs (green) in the ganglion cell layer (GCL) and the inner plexiform and inner nuclear layer (INL). ONL = outer nuclear layer. (**D**) High power images to show RTN3 protein localised to βIII-tubulin^+^ RGCs (white arrows) in the GCL. (**E**) GAP43 immunohistochemistry (* = lesion site) and (**F**) quantification to show that in control optic nerve, few, if any, GAP43^+^ axons pass beyond the lesion site, whilst in RTN3 overexpressed eyes, significant GAP43^+^ axons are present beyond the lesion site, an effect which is obliterated when Protrudin is knocked out at the same time as RTN3 overexpression. (**G**) Representative images to show FluorGold (FG) backfilled RGC in retinal wholemounts after RTN3 overexpression. (**H**) Quantification of the number of FG^+^ RGC in retinal wholemounts shows that overexpression of RTN3 is significantly neuroprotective, an effect that is independent of protrudin. (**I**) Representative ERG traces and (**J**) quantification of the pSTR amplitude show significant improvements in RTN3 overexpressed eyes, which are ablated after protrudin knockdown. Black arrows show the peak of the pSTR. Data are means ± SEM. *n* = 12 eyes/optic nerves/treatment. Scale bars in (**C**) = 100 µm, scale bars in (**D**) = 25 µm, scale bars in (**E**) = 200 µm, scale bars in (**G**) = 50 µm. *** *p* = 0.0001, one-way ANOVA with Dunnett’s post hoc test.

## References

[1] Alhajlah S., Thompson A.M., Ahmed Z. (2021). Overexpression of Reticulon 3 Enhances CNS Axon Regeneration and Functional Recovery after Traumatic Injury. Cells.

[2] Vigneswara V., Berry M., Logan A., Ahmed Z. (2013). Pigment Epithelium-Derived Factor Is Retinal Ganglion Cell Neuroprotective and Axogenic After Optic Nerve Crush Injury. Invest. Ophthalmol. Vis. Sci..

