# Supplementary material for: Overexpression of Reticulon 3 Enhances CNS Axon Regeneration and Functional Recovery after Traumatic Injury
Source: Cells. 2021 Aug 6;10(8):2015. doi: 10.3390/cells10082015 (PMC8395006; doi:10.3390/cells10082015)
Supplement: Supplementary file 1 [file cells-10-02015-s001.zip › SUPPLEMENTARY MATERIALS.pdf]

SUPPLEMENTARY MATERIALS

Overexpression of Reticulon 3 enhances CNS axon regeneration and functional recovery after injury

Sharif Alhajlah[1,2], Adam M. Thompson[1], Zubair Ahmed[1]*

[1]Neuroscience and Ophthalmology, Institute of Inflammation and Ageing, University of Birmingham, Edgbaston, Birmingham, B15 2TT, UK.
[2]Applied Medical Science College, Shaqra University, Addawadmi, Riyadh, Saudi Arabia.

Supplementary Materials

Figure S1. Knockdown of RTN3 mRNA by increasing amount of PEI-shRTN3 plasmid DNA in DRGN cultures.

Figure S2. Overexpression of RTN3 and challenge with Aβ1-42 does not have any adverse effects on DRGN neurite outgrowth or survival.

Figure S3. Off-target gene expression is absent in RTN3-overexpressed DRGN treated with siRNAs.

Figure S4. Overexpression of RTN3 and knockdown of Protrudin in DRGN in vivo.

Figure S5. Overexpression of RTN3 in DRGN in vivo upregulates regeneration associated gene (RAGs) expression.

Figure S6. RTN3 overexpression in retinal cultures promotes RGC survival and neurite outgrowth in vitro, which is dependent on protrudin.

Supplementary Tables

Table S1. Primers used in this study for qRT-PCR.

Rat gene	Forward (5'-3')	Reverse (5'-3')
TNF-α	ACCACGCTCTTCTGTCTACTG	CTTGGTGGTTTGCTACGAC
IFN-γ	AGGATGCATTCATGAGCATCGCC	CACCGACTCCTTTTCCGCTTCCT
IL-6	TCTCTCCGCAAGAGACTTCCA	ATACTGGTCTGTTGTGGGTGG
IL-12	AATGTTCCAGTGCCTCAACCA	GATCAATCTCTTCGGAAGTGCA
IL-1β	GCAATGGTCGGGACATAGTT	AGACCTGACTTGGCAGAGGA
IFN-β	CGTTCCTGCTGTGCTTCTC	TGTAACTCTTCTCCATCTGTGAC
MX1	AACCCTGCTACCTTTCAA	AAGCATCGTTTTCTCTATTTC
IFIT	CTGAAGGGGAGCGATTGATT	AACGGCACATGACCAAAGAGTAGA
OAS1	TTCTACGCCAATCTCATCAGTG	GGTCCCCCAGCTTCTCCTTAC
Casp7	CAACGACACCGACGCTAATC	GGTCCTTGCCATGCTCATTC
GAPDH	AGACAGCCGCATCTTCTTGT	CTTGCCGTGGGTAGAGTCAT

Table S2. Pre-validated primers used in this study for qRT-PCR

Rat gene	Rn no/Cat no.	Manufacturer
RTN3	Rn01498010_m1<br>Cat no. 4351372	ThermoFisher Scientific,<br>Loughborough, UK
Protrudin	Rn01766356_m1<br>Cat no. 4331182	ThermoFisher Scientific,<br>Loughborough, UK
FYCO1	Rn01445996_m1<br>Cat no. 4351372	ThermoFisher Scientific,<br>Loughborough, UK
RAB7	Rn00576640_m1<br>Cat no. 4331182	ThermoFisher Scientific,<br>Loughborough, UK
SYT7	Rn00572234_m1<br>Cat no. 4331182	ThermoFisher Scientific,<br>Loughborough, UK

Supplementary Figures

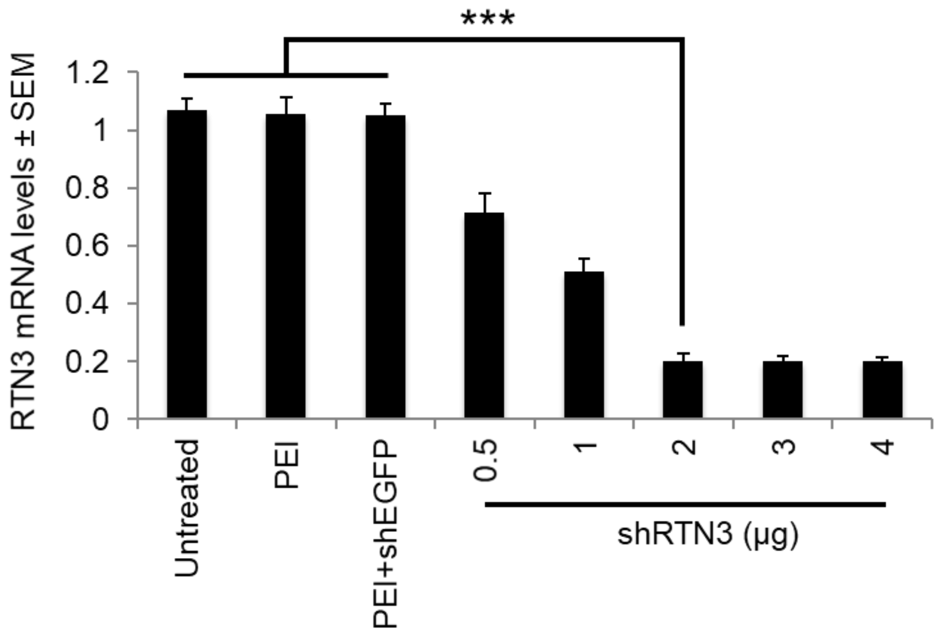

Figure S1: Knockdown of RTN3 mRNA by increasing amount of PEI-shRTN3 plasmid DNA in DRGN cultures. We determined that 2 µg of shRTN3 plasmids were required for maximal RTN3 knockdown. Data are means ± SEM. n = 9 wells/treatment. ***P = 0.0001, one-way ANOVA with Dunnett's post hoc test.

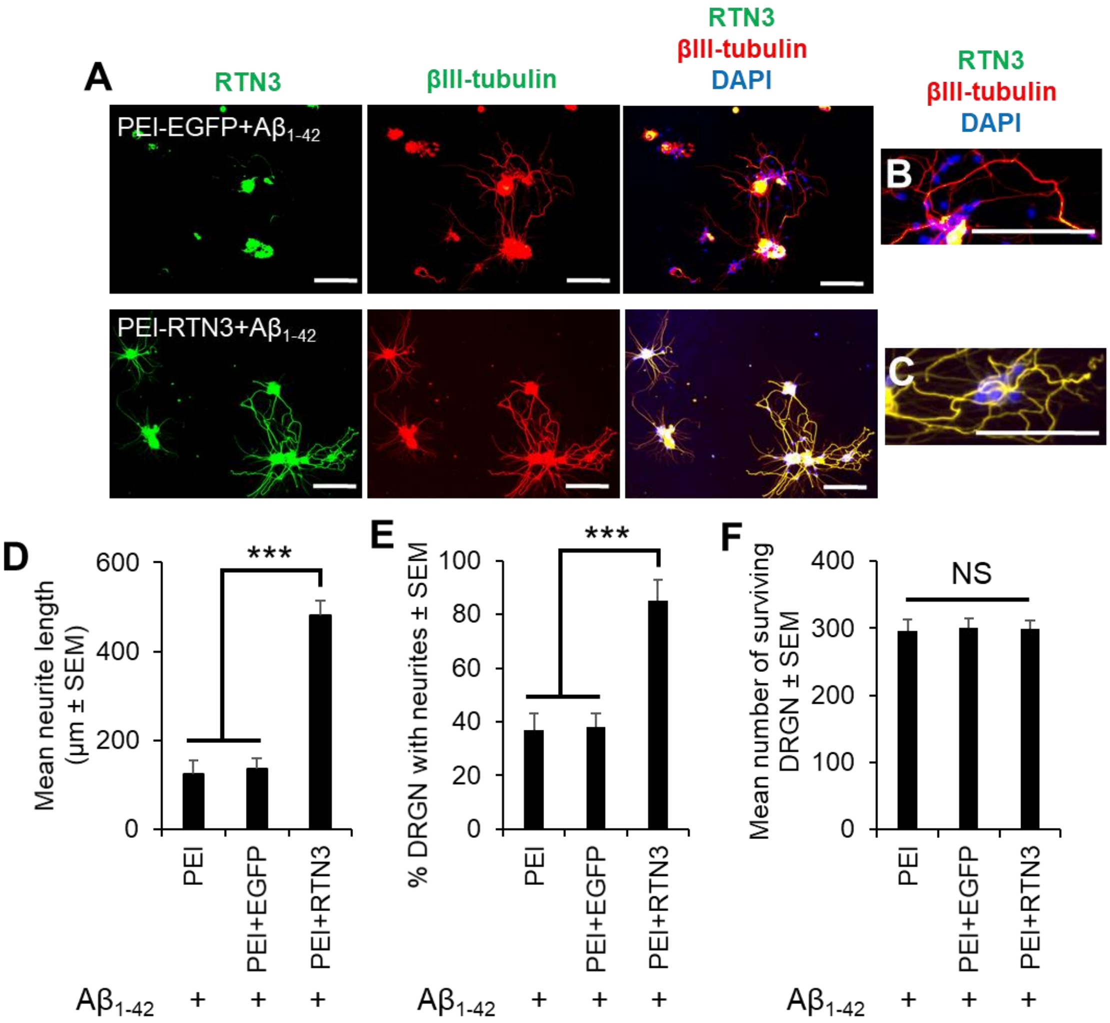

Figure S2: Overexpression of RTN3 and challenge with Aβ1-42 does not have any adverse effects on DRGN neurite outgrowth or survival. (**A**) Immunostaining for RTN3 and βIII-tubulin in DRGN cultures, challenged with Aβ1-42 to show both upregulation of RTN3 in DRGN cultures and normal neurite outgrowth. High power images to show normal appearance of neurites in (**B**) control and (**C**) RTN3 overexpressed DRGN. (**D**) The mean neurite length, (**E**) % DRGN with neurites and (**F**) DRGN survival are unaffected by Aβ1-42 exposure. Scale bars in (A) = 50 µm, scale bars in (B and C) = 200 µm. ***P = 0.0001, one-way ANOVA with Dunnett's post hoc test.

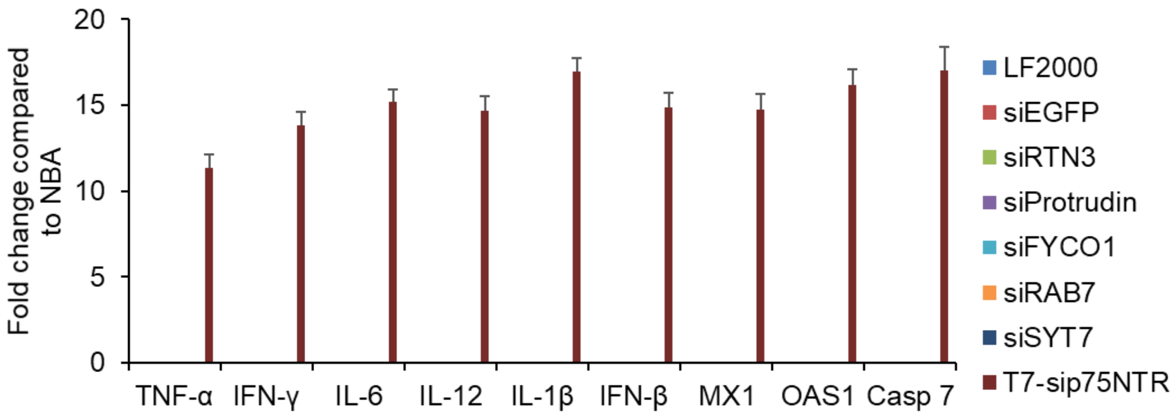

Figure S3: Off-target gene expression is absent in RTN3-overexpressed DRGN treated with siRNAs. qRT-PCR shows lack of activation of interferon-related or innate immunity-related genes after siRNA transfections in RTN3-overexpressed DRGN. The positive control sequence (sip75^{NTR}; Read et al., 2009) invokes 12-17-fold changes in all of these genes. Data are means ± SEM. *n* = 6 wells/treatment.

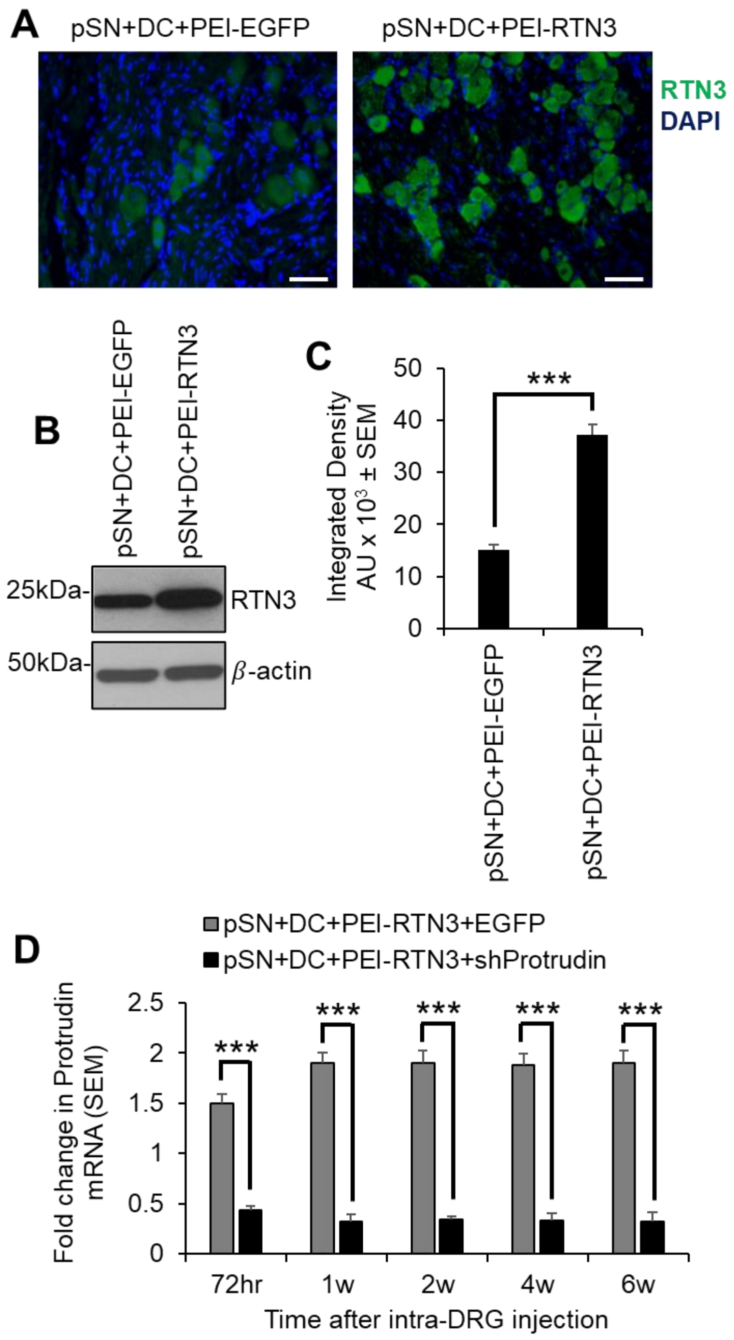

Figure S4: Overexpression of RTN3 and knockdown of Protrudin in DRGN in vivo. (**A**) Representative immunohistochemistry to show RTN3 immunoreactivity in control (pSN+DC+PEI-EGFP) and after overexpression of RTN3 in pSN+DC+PEI-RTN3-treated animals. (**B**) Western blot to show levels of RTN3 protein after overexpression of RTN3. β-actin is used as a protein loading control. (**C**) Densitometry showed significant upregulation of RTN3 protein after overexpression using RTN3 plasmids. (**D**) qRT-PCR to show knockdown in protrudin mRNA over time after in vivo DRG injection PEI-shProtrudin. Data are means ± SEM. *n* = 18 rats/group/test. Scale bars in (A) = 50µm. ***P = 0.0001, one-way ANOVA with Dunnett's post hoc test.

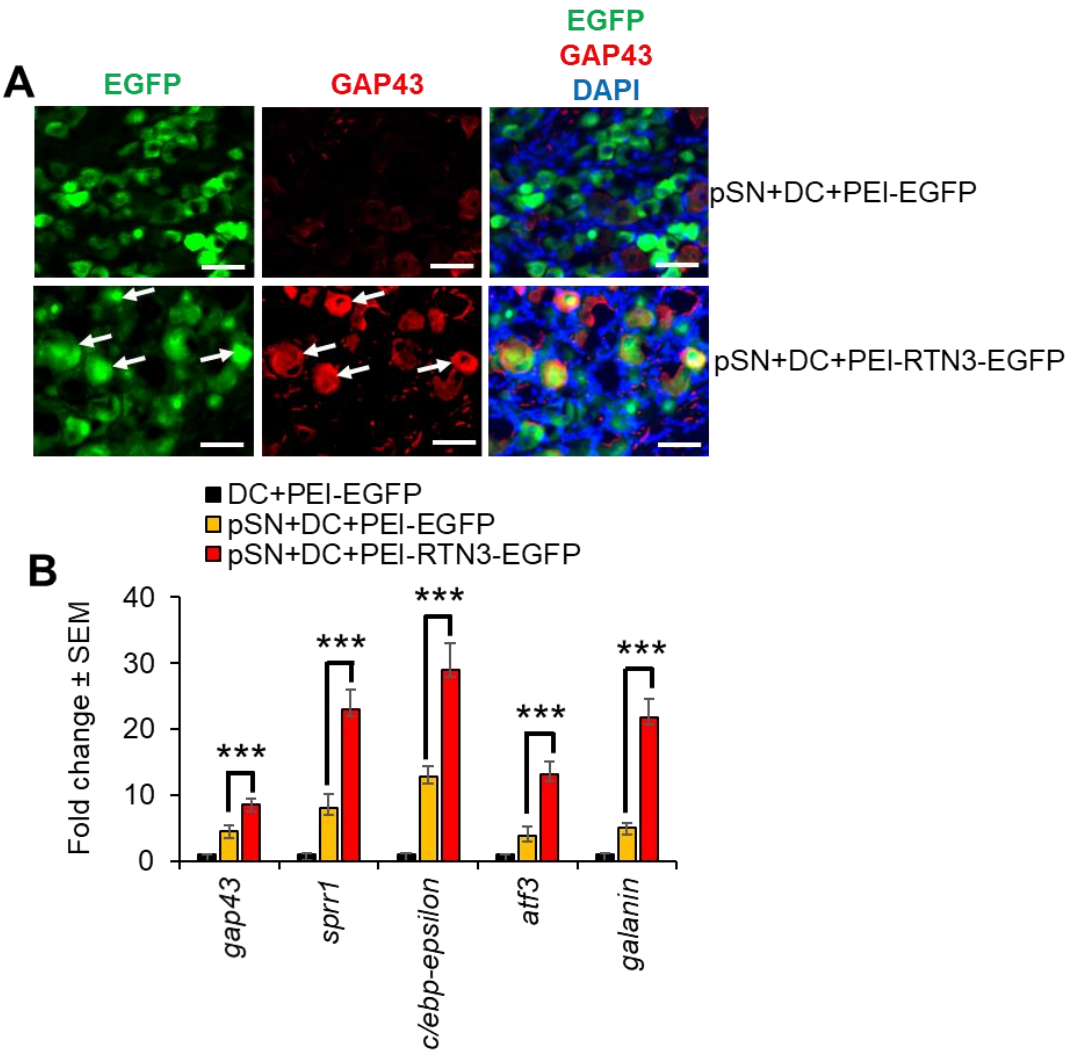

Figure S5: Overexpression of RTN3 in DRGN in vivo upregulates regeneration associated gene (RAGs) expression. (**A**) Immunohistochemistry to show induction of $GAP43^{+}$ immunoreactivity in RTN3 overexpressed DRGN (as measured by the reporter gene EGFP in green; arrows). (**B**) qRT-PCR to demonstrate upregulation of RAGs (*gap43, sprr1a, cebp-epsilon, atf3 and galanin*) after preconditioning lesions and further upregulation after RTN3 overexpression. Data are means ± SEM. *n* = 12 rats/group/test. Scale bars in (A) = 25µm. ***P = 0.0001, one-way ANOVA with Dunnett's post hoc test.

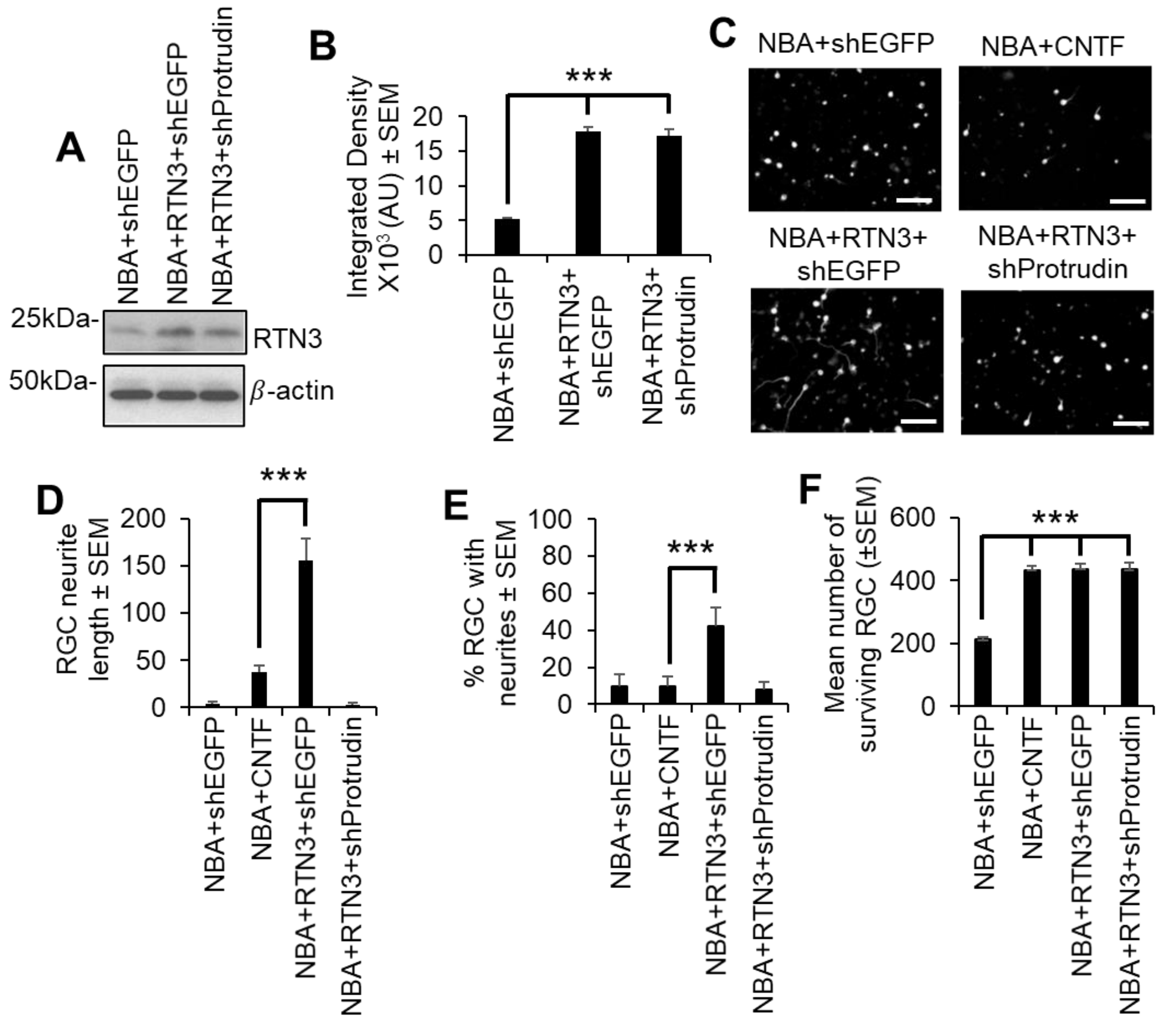

Figure S6: RTN3 overexpression in retinal cultures promotes RGC survival and neurite outgrowth in vitro, which is dependent on protrudin. (**A**) Western blot to show that RTN3 is significantly overexpressed in retinal cells treated with RTN3 plasmids. (**B**) Densitometry shows greater than 3-fold upregulation of RTN3 protein levels in retinal cultures by RTN3 plasmids. (**C**) Representative images stained with βIII-tubulin and (**D**) quantification of the mean neurite length and (**E**) % RGC with neurites to show that RTN3 overexpression increased neurite length and the number of RGC with neurites, an effect which was ablated after knockdown of Protrudin. (**F**) RTN3 overexpression promotes significant RGC survival, which was not affected by protrudin knockdown. Data are means ± SEM. *n* = 9 wells/treatment. Scale bars in (C) = 100µm; ***P=0.0001, one-way ANOVA with Dunnett's post hoc test.
